# A cross-sectional study on the impact of physical exercise on mobile phone addiction: the chain mediating role of perceived stress and self-acceptance

**DOI:** 10.3389/fpsyg.2025.1671549

**Published:** 2025-09-18

**Authors:** Zhen Ding, Xinru Qi, Tianle Fang, Lishun Xiao, Dehui Yin, Zhiming Sun

**Affiliations:** ^1^School of Public Health, Xuzhou Medical University, Xuzhou, China; ^2^Office of the President, Xuzhou Medical University, Xuzhou, China

**Keywords:** physical exercise, mobile phone addiction, perceived stress, self- acceptance, chain mediation

## Abstract

**Objective:**

This study aims to explore the potential pathways underlying the association between physical exercise and mobile phone addiction among university students, with particular attention to the possible sequential mediating roles of perceived stress and self-acceptance. The goal is to generate insights that may inform future research and contribute to a deeper understanding of psychological health in higher education settings.

**Methods:**

This cross-sectional study employed a convenience sampling method to survey 1,392 undergraduate students from Xuzhou Medical University. The study utilized the Physical Activity Rating Scale (PARS-3), the Mobile Phone Addiction Tendency Scale (MPATS), the Perceived Stress Scale (PSS), and the Self-Acceptance Questionnaire (SAQ). Data were analyzed using SPSS 27.0 and the PROCESS macro (Model 6) to conduct descriptive statistics, correlation analysis, and mediation analysis.

**Results:**

Physical exercise was significantly negatively correlated with mobile phone addiction (*r* = −0.293, *p* < 0.01) and perceived stress (*r* = −0.326, *p* < 0.01), and positively correlated with self-acceptance (*r* = 0.408, *p* < 0.01). The total effect of physical exercise on mobile phone addiction was −0.291, and the direct effect was −0.135. The indirect effect through perceived stress was −0.118 (95% CI: [−0.149, −0.090]), through self-acceptance was −0.024 (95% CI: [−0.041, −0.010]), and the chain mediating effect through both perceived stress and self-acceptance was −0.014 (95% CI: [−0.024, −0.006]). All indirect effects were statistically significant, indicating that physical exercise significantly affects mobile phone addiction through these pathways.

**Conclusion:**

Physical exercise has been found to show a negative association with mobile phone addiction among university students. This association appears to involve both direct links and indirect pathways through lower levels of perceived stress and higher levels of self-acceptance, suggesting a potential chain mediating relationship. These findings contribute to a deeper understanding of the complex interplay between physical activity, psychological factors, and mobile phone use behaviors in university populations.

## 1 Introduction

According to the 55th Statistical Report on Internet Development in China released by the China Internet Network Information Center (CNNIC) (Center CINI, 2025), as of 2024, the number of internet users in China reached 1.108 billion, with an internet penetration rate of 78.6%. With the rapid advancement of mobile internet technologies and the continuous evolution of smart devices, mobile phones have become an indispensable tool in the daily lives of contemporary university students (Zeerak et al., 2024). They are widely used for academic purposes, social interactions, entertainment, and emotional regulation (Li et al., 2024). Although such usage improves the efficiency of information acquisition and facilitates social connectivity, it has also led to a series of psychological and behavioral concerns (Zhang et al., 2021; Fruehwirth et al., 2024). Among these issues, the growing phenomenon of mobile phone addiction—also referred to as problematic smartphone use—has received increasing attention from both academia and the general public. Mobile phone addiction is characterized by a strong and persistent psychological dependence and behavioral compulsion toward mobile phone use, such that individuals continue using their devices excessively despite being aware of the negative consequences (Achangwa et al., 2022). Luo et al. (2025) found that mobile phone addiction is often accompanied by symptoms such as attention deficits, emotional instability, social withdrawal, heightened loneliness, and academic procrastination. These symptoms may further lead to psychological disorders such as anxiety, depression, and impulse control issues, thereby adversely affecting individuals’ quality of life, social adaptability, and mental wellbeing.

In the pursuit of effective intervention strategies, increasing attention has been directed toward understanding how physical exercise may mitigate mobile phone addiction. Physical activity has been shown to enhance psychological well-being, thus alleviating the emotional vulnerabilities that contribute to problematic smartphone use (Buke et al., 2021). A growing body of research indicates that regular aerobic exercise promotes relaxation, enhances feelings of pleasure and satisfaction, reduces anxiety and depression, improves sleep quality, and relieves everyday stress. These benefits collectively reduce individuals’ reliance on digital media for instant gratification and may lower the risk of mobile phone addiction (Mandolesi et al., 2018; Yang et al., 2021; Hossain et al., 2024). A cross-sectional study conducted among Chinese university students revealed that those who frequently participated in group physical activities and expressed a greater interest in sports spent less time on social media and reported higher levels of subjective well-being (Shi et al., 2025).

In exploring the mechanisms through which physical exercise influences mobile phone addiction, perceived stress has been widely identified as a key mediating psychological variable. Perceived stress is defined as the degree to which individuals appraise situations in their lives as stressful, unpredictable, uncontrollable, or overwhelming. It reflects the subjective experience of stress rather than the objective intensity of stressors and is closely linked to emotional and physiological responses (Padmanabhanunni et al., 2023). According to Lazarus and Folkman’s stress-coping theory, individuals perceive varying levels of stress in response to environmental stimuli based on their subjective evaluations. Higher levels of perceived stress are frequently associated with maladaptive coping behaviors, including various forms of addiction (Obbarius et al., 2021). Previous studies have demonstrated that perceived stress is a significant psychological predictor of mobile phone addiction (Gao et al., 2018; Zhang et al., 2022). For instance, Wang et al. (2021) found that among medical students—a population particularly susceptible to high stress—greater levels of perceived stress were associated with higher rates of excessive mobile phone use and stronger patterns of dependency. This suggests that under stress, individuals are more likely to seek temporary psychological relief through mobile phones, thereby increasing their addiction risk. Physical exercise, as an effective stress-regulating activity, has been shown to significantly reduce perceived stress levels (Hackney, 2006).

Another important psychological variable in this context is self-acceptance, defined as the comprehensive acknowledgment and acceptance of one’s strengths, weaknesses, and uniqueness. Self-acceptance is considered a key indicator of psychological health (Ruan et al., 2023). During physical activities, individuals may experience enhanced self-efficacy through positive bodily sensations (Tikac et al., 2022), while the social support and sense of belonging present in exercise environments help fulfill basic psychological needs (Murphy et al., 2022), thereby promoting higher levels of self-acceptance. Prior research suggests that individuals with low self-acceptance are more prone to experiencing negative emotions such as depression and anxiety (Sowislo and Orth, 2013). The relationship between self-acceptance and mobile phone addiction primarily lies in its role in reducing dependency on external virtual environments. Mobile phone addiction is often driven by a desire for social media validation and recognition of one’s virtual identity, behaviors typically rooted in low self-worth and emotional dysregulation (Andreassen et al., 2017, Wacks and Weinstein, 2021). Individuals with higher levels of self-acceptance are more likely to embrace their authentic selves and less likely to rely on external validation, thus reducing the tendency to use mobile phones as a means of emotional compensation or escapism (Guo et al., 2025).

The impact of perceived stress on psychological and behavioral outcomes does not occur in isolation; self-acceptance also plays a critical role in this dynamic. Emerging theoretical and empirical evidence suggests that perceived stress not only directly affects behavior but may also indirectly influence outcomes through its impact on self-acceptance levels (Yan et al., 2022). Specifically, high levels of perceived stress may undermine an individual’s ability to accept themselves, resulting in psychological maladjustment and emotional distress, which in turn exacerbates dependency on mobile phones and other virtual media, forming a negative feedback loop (Lee et al., 2019). This sequential mediating effect between perceived stress and self-acceptance provides a novel perspective for understanding the complex psychological mechanisms underlying smartphone addiction and offers theoretical grounding for intervention strategies centered on psychological regulation.

**Figure 1:**
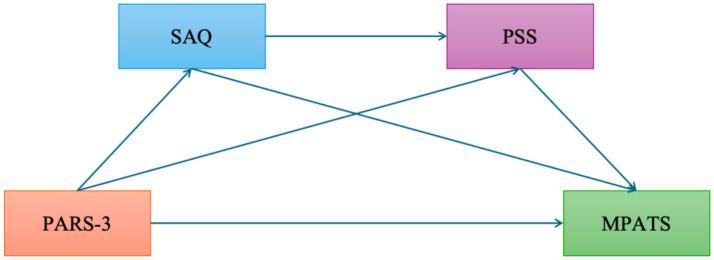
Hypothetical chain mediation model of physical exercise, self-acceptance, perceived stress, and mobile phone addiction. This figure illustrates the hypothesized relationships among physical exercise, self-acceptance, perceived stress, and mobile phone addiction. Arrows indicate the direction of influence. PARS-3, Physical Exercise, MPATS, Mobile phone Addiction; SAQ, Self-Acceptance; PSS, Perceived Stress.

Based on the above theoretical and empirical foundations, the present study proposes a chain mediation model (see Figure 1) to examine the mediating roles of perceived stress and self-acceptance in the relationship between physical exercise and smartphone addiction. The following hypotheses are proposed:

Hypothese 1: The frequency of physical exercise is negatively associated with mobile phone addiction among university students.

Hypothese 2: Perceived stress mediates the relationship between physical exercise and mobile phone addiction.

Hypothese 3: Self-acceptance mediates the relationship between physical exercise and mobile phone addiction.

Hypothese 4: Perceived stress and self-acceptance jointly mediate the relationship between physical exercise and mobile phone addiction through a chain mediation pathway.

## 2 Methods

This study employed a cross-sectional design to investigate the relationship between physical exercise and mobile phone addiction among university students, with perceived stress and self-acceptance as potential mediators. A convenience sampling method was used to survey undergraduate students of Xuzhou Medical University. The study utilized the following instruments: the Physical Activity Rating Scale (PARS-3), the Mobile Phone Addiction Tendency Scale (MPATS), the Perceived Stress Scale (PSS), and the Self-Acceptance Questionnaire (SAQ). All scale scores were treated as continuous variables in the analysis. Data were analyzed using SPSS 27.0 and the PROCESS macro (Model 6) to conduct descriptive statistics, correlation analysis, and mediation analysis.

### 2.1 Participants

This study employed a convenience sampling method, targeting undergraduate students from Xuzhou Medical University. To avoid overgeneralization, it should be emphasized that the sample was limited to students of Xuzhou Medical University and may not represent the broader college student population. All research procedures were conducted in strict accordance with the principles of the Declaration of Helsinki, relevant regulations, and institutional ethical standards. Participants were invited to complete the questionnaire through the online survey platform “Wenjuanxing.” To ensure data quality, valid responses were defined as those with complete demographic information and no missing key questionnaire items. Questionnaires were excluded if they contained missing demographic data, excessive item nonresponse, or showed inconsistent answering patterns. All data were screened based on these criteria prior to analysis. To reduce common method bias, anonymity and confidentiality were assured to minimize social desirability effects.

### 2.2 Measures

#### 2.2.1 Physical activity rating scale (PARS-3)

This study adopted the Physical Activity Rating Scale (PARS-3) revised by Chinese scholar Liang Deqing (Liang, 1994). The scale evaluates an individual’s level of physical activity across three dimensions: intensity, duration, and frequency, with each dimension scored on a 1 to 5 scale. The final physical activity score is calculated using the following formula: Physical Activity Level = Intensity × (Duration – 1) × Frequency. The physical activity score ranges from 0 to 100, with low level of physical exercise ≤19 points, medium level of physical exercise with 20–42 points, and high level of physical exercise ≥43 points. Higher scores indicate more positive views and higher willingness to engage in regular exercise. These scores help understand motivation and readiness for physical activity interventions. The scale demonstrated good reliability, with a Cronbach’s alpha coefficient of 0.802.

#### 2.2.2 Mobile phone addiction tendency scale (MPATS)

The MPATS was developed by Xiong et al. (2012). It consists of 16 items rated on a 5-point Likert scale ranging from “strongly disagree” to “strongly agree.” The scale comprises four dimensions: withdrawal symptoms, salience behavior, social comfort, and mood changes. The total score ranges from 16 to 80, with higher scores indicating a greater tendency toward mobile phone addiction. Scores below 47 indicate normal use, while scores of 48 and above suggest the presence of dependence. The scale demonstrated high reliability, with a Cronbach’s alpha coefficient of 0.932.

#### 2.2.3 Perceived stress scale (PSS)

The Chinese version of the Perceived Stress Scale was developed by Yang and Huang (2003). It consists of 14 items divided into two dimensions: sense of helplessness and sense of tension, each comprising 7 items. The scale uses a 5-point Likert format, with higher total scores indicating greater perceived stress. It is widely used to screen for stress-related health risks in both clinical and general populations. The scale demonstrated good reliability, with a Cronbach’s alpha coefficient of 0.810.

#### 2.2.4 Self-acceptance questionnaire (SAQ)

The SAQ was developed by Cong and Gao (1999). It comprises 16 items divided into two dimensions: self-acceptance and self-evaluation, each containing 8 items. The scale uses a 4-point Likert format. Items in the self-acceptance dimension are reverse-scored, and higher total scores indicate a greater level of self-acceptance. It reflects one’s psychological well-being and self-concept stability. The questionnaire demonstrates good reliability, with a Cronbach’s alpha coefficient of 0.837.

### 2.3 Statistical analysis

Data processing and analysis were conducted using SPSS version 27.0 and the PROCESS macro. Descriptive statistics, independent sample t-tests, and analysis of variance (ANOVA) were used to examine differences across demographic variables. Pearson correlation analysis was employed to assess the relationships among the main variables. Mediation and chained mediation effects were tested using regression analysis combined with the Bootstrap method, with 5,000 resamples and a 95% confidence interval. All hypothesis testing was conducted using two-tailed tests, with a significance level set at *p* < 0.05. Mediation analyses were performed based on Model 6 of the PROCESS macro developed by Hayes. Gender, household registration type, only-child status, and monthly living expenses were included as control variables to account for potential confounding effects. To assess potential common method bias, Harman’s single-factor test was conducted.

## 3 Results

### 3.1 Control and inspection of common method deviation

Harman’s single-factor test revealed that nine factors had eigenvalues greater than 1, with the first factor accounting for 28.340% of the total variance, which is below the critical threshold of 40%. These findings suggest that common method bias is not a serious concern in this study.

### 3.2 Participant selection process

**Figure 2:**
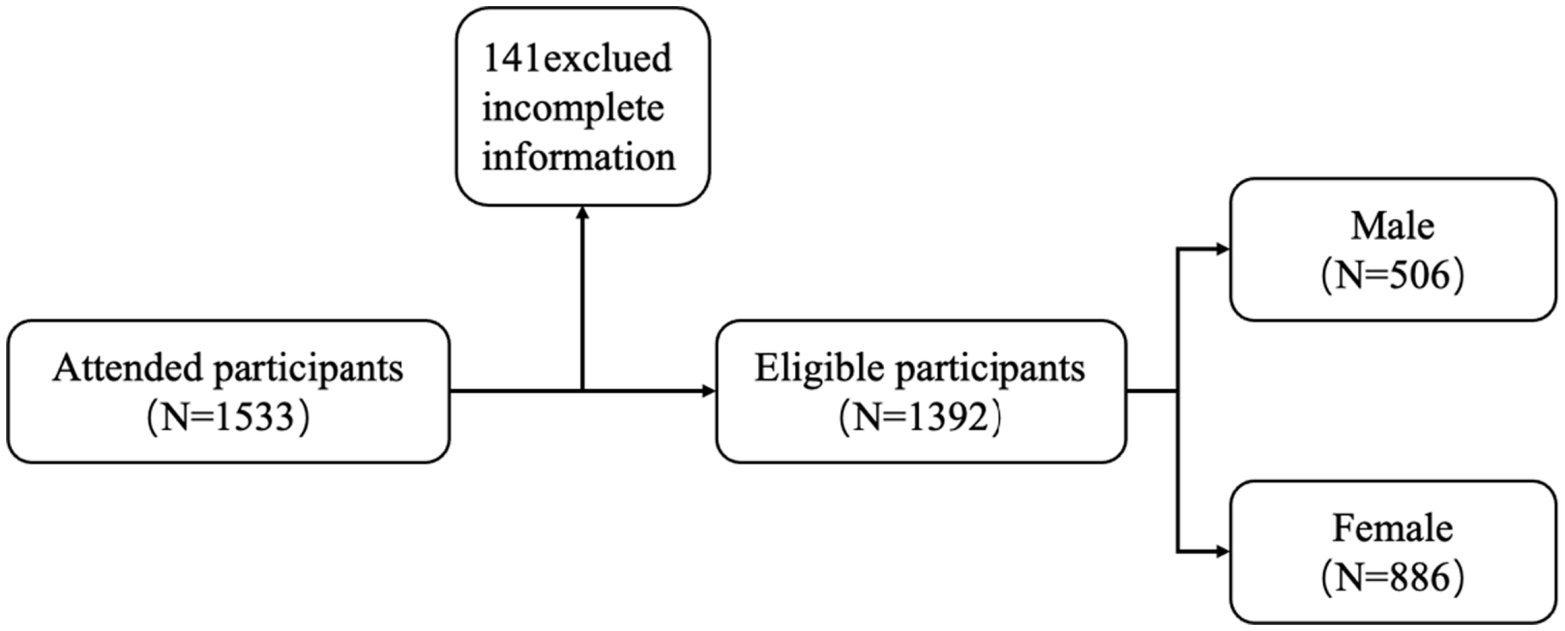
Flowchart of study participants.

To ensure data quality, rigorous screening procedures were implemented. A total of 1,533 questionnaires were distributed, and following the exclusion of 141participants due to incomplete demographic information or invalid questionnaire responses, a final sample of 1,392 valid participants was retained for statistical analysis (see Figure 2).

### 3.3 Participant characteristics

Table 1 presents an overview of their demographic characteristics. Among the participants, 506 were male (36.4%) and 886 were female (63.6%). Urban residents accounted for 639 individuals (45.90%), while 753 (54.10%) were from rural areas. A total of 534 participants (38.34%) were only children, and 858 (61.66%) were non-only children. Regarding monthly living expenses, 60 participants (4.31%) reported expenses below 1,000 RMB; 977 (70.19%) reported 1,001–2,000 RMB; 315 (22.63%) reported 2,001–3,000 RMB; and 40 (2.87%) reported more than 3,000 RMB. Additionally, a significant difference in physical activity levels was observed across gender, and perceived stress levels varied significantly by monthly living expenses (see Table 1).

**Table 1:** Demographic information and variable differences.

Variables		N (%)	PARS-3 (M ± SD)	MPATS (M ±SD )	SAQ (M ± SD)	PSS (M ± SD)
Gender	Male	506	21.92 ± 21.665	2.655 ± 0.753	2.374 ± 0.510	2.767 ± 0.701
	Female	886	23.93 ± 22.787	2.598 ± 0.710	2.438 ± 0.490	2.754 ± 0.649
*t*			−1.626	1.408	−2.286	0.335
*p*			*	0.404	0.428	0.100
Native place	Urban	639	22.48 ± 21.849	2.631 ± 0.733	2.406 ± 0.502	2.788 ± 0.678
	Rural	753	23.81 ± 22.851	2.608 ± 0.721	2.422 ± 0.496	2.734 ± 0.660
*t*			−1.109	0.590	−0.613	1.506
*p*			0.217	0.764	0.790	0.915
Only child or not	Yes	534	22.67 ± 22.175	2.685 ± 0.726	2.381 ± 0.504	2.763 ± 0.665
	No	858	23.53 ± 22.543	2.578 ± 0.724	2.436 ± 0.494	2.756 ± 0.671
*t*			−0.691	2.682	−2.009	0.188
*p*			0.725	0.593	0.806	0.697
Monthly living expenses	<1,000	60	18.78 ± 19.875	2.655 ± 0.641	2.473 ± 0.507	2.927 ± 0.635
	1,001–2000	977	23.42 ± 22.440	2.605 ± 0.730	2.411 ± 0.499	2.731 ± 0.657
	2001–3,000	315	23.57 ± 23.397	2.645 ± 0.728	2.410 ± 0.495	2.824 ± 0.705
	>3,000	40	21.35 ± 15.853	2.706 ± 0.754	2.440 ± 0.521	2.659 ± 0.633
*F*			0.930	0.510	0.331	3.142
*p*			0.425	0.675	0.803	*
Smoke	Yes	11	16.91 ± 16.718	2.688 ± 0.800	2.358 ± 0.509	3.006 ± 0.588
	No	1,381	23.25 ± 22.435	2.618 ± 0.726	2.415 ± 0.498	2.757 ± 0.669
*t*			−0.935	0.314	−0.378	1.236
*p*			0.061	0.872	0.852	0.522
Drink	Yes	105	23.18 ± 20.278	2.656 ± 0.699	2.424 ± 0.479	2.852 ± 0.677
	No	1,287	23.20 ± 22.570	2.616 ± 0.729	2.414 ± 0.500	2.751 ± 0.667
*t*			−0.008	0.544	0.210	1.497
*p*			0.293	0.619	0.615	0.690

PARS-3, Physical Exercise; MPATS, Mobile phone Addiction; SAQ, Self-Acceptance; PSS, Perceived Stress. *p < 0.05, **p < 0.01, ***p < 0.001. Mean values represent average (unstandardized) scores for each scale.

### 3.4 Correlation analysis

A correlation analysis was conducted to examine the relationships among mobile phone addiction, physical activity, self-acceptance, and perceived stress. As shown in Table 2, physical activity was positively correlated with self-acceptance (*r* = 0.408, *p* < 0.01), and negatively correlated with perceived stress (*r* = −0.326, *p* < 0.01) as well as mobile phone addiction (*r* = −0.293, *p* < 0.01). In addition, self-acceptance was negatively correlated with perceived stress (*r* = −0.380, *p* < 0.01), while perceived stress was positively correlated with mobile phone addiction (*r* = 0.273, *p* < 0.01).

**Table 2:** Correlation between variables.

Variables	M ± SD	PARS-3	MPATS	SAQ	PSS
PARS-3	23.20 ± 22.399	1			
MPATS	2.619 ± 0.726	−0.293**	1		
SAQ	2.415 ± 0.498	0.408**	−0.394**	1	
PSS	2.759 ± 0.668	−0.326**	0.273**	−0.380**	1

PARS-3, Physical Exercise; MPATS, Mobile phone Addiction; SAQ, Self-Acceptance; PSS, Perceived Stress. *p < 0.05, **p < 0.01. Mean values represent average (unstandardized) scores for each scale.

### 3.5 Regression and mediation analysis

Stepwise regression analysis was conducted to examine both the direct and indirect effects of physical activity on mobile phone addiction. As shown in Table 3, physical activity significantly and positively predicted self-acceptance (*β* = 0.406, *p* < 0.001), and significantly and negatively predicted perceived stress (*β* = −0.205, *p* < 0.001), mobile phone addiction (*β* = −0.291, *p* < 0.001), and the chained mediation pathway leading to mobile phone addiction (*β* = −0.135, *p* < 0.001). These findings suggest that physical activity contributes to higher levels of self-acceptance, reduced perceived stress, and lower risk of mobile phone addiction. Furthermore, self-acceptance significantly and negatively predicted both perceived stress (*β* = −0.298, *p* < 0.001) and mobile phone addiction (*β* = −0.291, *p* < 0.001), while perceived stress significantly and positively predicted mobile phone addiction (*β* = 0.119, *p* < 0.001).

**Table 3:** Mediating effects in the regression model of physical activity and mobile phone addiction.

Variables	MPATS			SAQ			PSS			MPATS		
	β	*t*	*p*	β	*t*	*p*	β	*t*	*p*	β	*t*	*p*
Gender	−0.018	−0.697	0.486	0.039	1.571	0.117	0.016	0.666	0.506	−0.007	−0.299	0.765
Native place	0.018	0.660	0.509	−0.012	−0.472	0.637	−0.038	−1.437	0.151	0.019	0.715	0.475
Only child or not	−0.066	−2.382	0.017	0.044	1.664	0.096	0.025	0.955	0.340	−0.055	−2.085	0.037
Monthly living expense	0.015	0.546	0.585	−0.005	−1.187	0.852	0.002	0.088	0.930	0.013	0.506	0.613
PARS-3	−0.291	−11.357	<0.001	0.406	16.573	<0.001	−0.205	−7.689	<0.001	−0.135	−4.968	<0.001
SAQ							−0.298	−11.159	<0.001	−0.291	−10.484	<0.001
PSS										0.119	4.456	<0.001
R	0.301			0.413			0.426			0.437		
�R ^2^	0.091			0.170			0.181			0.191		
*F*	27.656***			56.908***			51.109***			46.541***		

PARS-3, Physical Exercise; MPATS, Mobile phone Addiction; SAQ, Self-Acceptance; PSS, Perceived Stress. *p < 0.05, **p < 0.01, ***p < 0.001.

**Figure 3:**
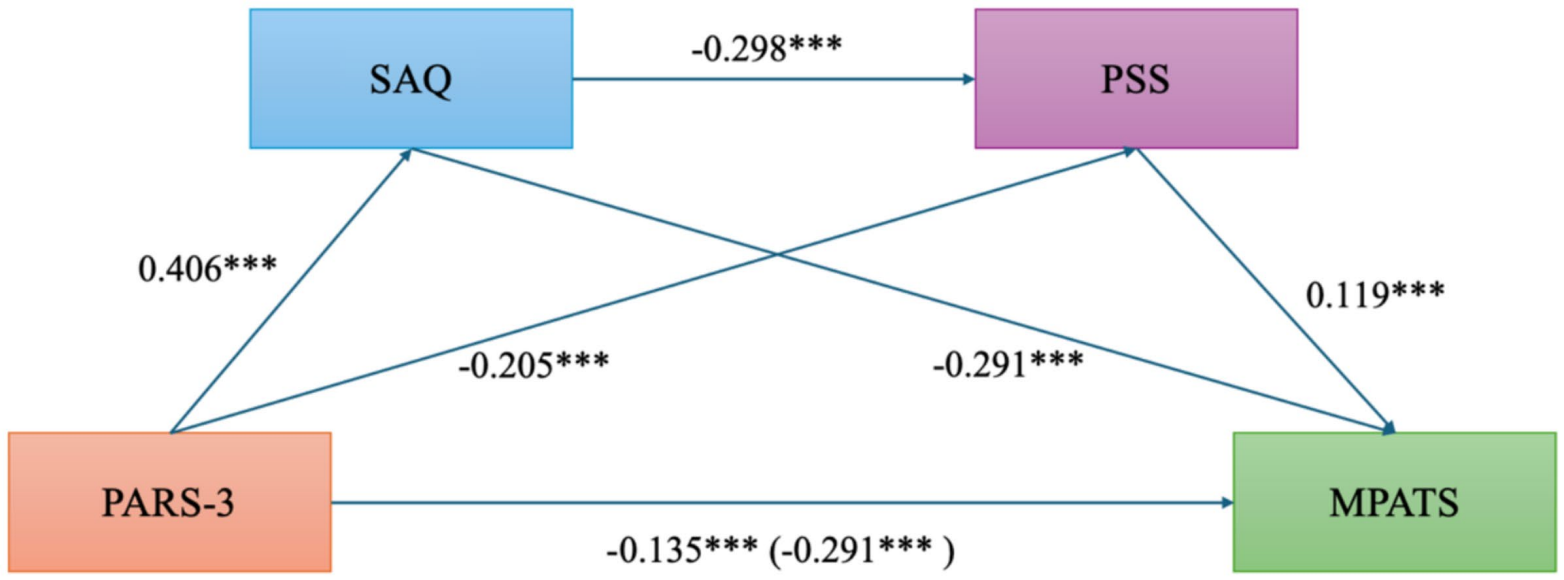
Mediation model of self-acceptance and perceived stress in the relationship between physical activity and mobile phone addiction. PARS-3, Physical Exercise; MPATS, Mobile phone Addiction; SAQ, Self-Acceptance; PSS, Perceived Stress. *p < 0.05 **p < 0.01 ***p < 0.001.

Quantitative analysis of the mediation effects (as shown in Table 4 and Figure 3) revealed that the total effect of physical activity on mobile phone addiction was −0.291, with a direct effect of −0.135. The 95% confidence intervals for both effects did not include zero, indicating a significant negative impact of physical activity on mobile phone addiction. The indirect effect through perceived stress was −0.118 (95% CI: [−0.149, −0.090]), the indirect effect through self-acceptance was −0.024 (95% CI: [−0.041, −0.010]), and the chained mediation effect via both perceived stress and self-acceptance was −0.014 (95% CI: [−0.024, −0.006]). All confidence intervals excluded zero, indicating statistical significance. The total indirect effect was −0.157 (95% CI: [−0.188, −0.127]), suggesting that perceived stress and self-acceptance partially mediate the relationship between physical activity and mobile phone addiction.

**Table 4:** Mediation effects of physical activity on mobile phone addiction.

Benefit type	Effect value	BootSE	Bootstrap 95%CI	
			Boot LLCI	Boot ULCI
Total effect	−0.291	0.0008	−0.011	−0.008
Direct effect	−0.135	0.0009	−0.006	−0.002
Indirect effect 1	−0.118	0.015	−0.149	−0.090
Indirect effect 2	−0.024	0.008	−0.041	−0.010
Indirect effect 3	−0.014	0.004	−0.024	−0.006
Total indirect effect	−0.157	0.016	−0.188	−0.127

## 4 Discussion

With the widespread use of smartphones, mobile phone addiction has increasingly become a pressing mental health concern among university students worldwide (Shang et al., 2024). A growing body of research has shown that excessive reliance on mobile phones not only leads to academic underperformance and attention deficits but also contributes to various psychological disorders such as anxiety and depression, thereby severely impacting individuals’ quality of life and social adaptability (Alavi et al., 2020, Abuhamdah and Naser, 2023, Datta et al., 2025). In light of this, identifying strategies that are associated with lower levels of mobile phone addiction among university students has emerged as a critical issue in the fields of social psychology, education, and public health. Based on a sample of 1,392 university students, the present study systematically examined the role of physical activity in alleviating mobile phone addiction and explored its underlying psychological mechanisms, thereby providing valuable empirical evidence for the field.

The study found a significant negative correlation between physical activity and mobile phone addiction: higher frequency of physical activity was associated with lower levels of mobile phone addiction among university students (*r* = −0.293, *p* < 0.01), supporting Hypothesis 1. This finding is consistent with previous research (Zhao et al., 2022). Physical activity promotes the release of neurotransmitters such as endorphins and dopamine, which enhance positive emotional experiences and psychological satisfaction. As a result, individuals individuals may show less reliance on the immediate gratification associated with mobile phone use, which is possibly related to both physiological and psychological processes (Chen et al., 2022). The neurochemical changes induced by physical activity not only alleviate negative emotions but also strengthen cognitive control, thereby improving individuals’ ability to regulate their mobile phone use. In practice, regular physical activity can effectively occupy one’s leisure time, serving as a behavioral substitute that reduces fragmented and impulsive phone use (Meng et al., 2025). Therefore, physical activity is not only a healthy lifestyle choice but also functions as an effective behavioral alternative, playing a positive role in the prevention and mitigation of mobile phone addiction.

Further analysis revealed support for Hypothesis 2, indicating that perceived stress significant mediates the association between physical activity and mobile phone addiction. Research data showed that the indirect effect through perceived stress was −0.118, with a 95% confidence interval of [−0.149, −0.090], which does not include zero and thus confirms statistical significance. In the biological mechanisms underlying depression, dysfunction of the hypothalamic–pituitary–adrenal (HPA) axis has been identified as a key pathological factor in the onset and progression of the disorder (Sharan and Vellapandian, 2024). As a subjective psychological factor, perceived stress may trigger long-term dysregulation of the HPA axis and is widely regarded as a high-risk factor for the onset and exacerbation of depressive symptoms (Mariani et al., 2023). Physical activity serves as a positive emotion regulation strategy; frequent engagement in physical activity has been associated with HPA axis balance and lower cortisol levels, thereby effectively alleviating psychological stress (Moyers and Hagger, 2023). As perceived stress decreases, individuals show less tendency to seek instant gratification or escape through mobile phone use (Zhang and Wang, 2022). These findings not only confirm the stress-buffering effect of physical activity in the context of addiction intervention but also provide practical implications for incorporating physical activity into campus-based mental health promotion efforts.

Research results confirmed Hypothesis 3, showing that self-acceptance significantly mediated the relationship between physical activity and mobile phone addiction. The indirect effect via self-acceptance was −0.024 (95% CI: [−0.041, −0.010]), with the confidence interval excluding zero, thereby indicating a statistically significant effect. Furthermore, physical activity was found to positively predict self-acceptance (*β* = 0.406, *p* < 0.001), which in turn negatively predicted mobile phone addiction (*β* = −0.291, *p* < 0.001), supporting the proposed mediation pathway. This suggests that engaging in physical activity enhances self-efficacy, bodily control, and positive social feedback, thereby fostering greater acceptance and affirmation of one’s self-integrity (Gong et al., 2023). In this process, individuals become less dependent on external validation and approval from virtual environments, leading to a reduction in compensatory mobile phone overuse (Chen et al., 2023). This finding aligns closely with existing theories that associate dependence on virtual identities with underlying deficits in self-identity (Pogorelov and Rylskaya, 2022). It also highlights the need for universities to integrate psychological education aimed at promoting self-identity and self-esteem into physical activity interventions to enhance their overall effectiveness.

Hypothesis 4 was supported through the chained mediation model, indicating that physical activity influences mobile phone addiction among university students via the sequential mediating effects of perceived stress and self-acceptance. The results showed a statistically significant chained indirect effect of −0.014 (95% CI: [−0.024, −0.006]), with the confidence interval excluding zero. The results of the chained mediation analysis indicate that physical activity first alleviates perceived stress, which subsequently enhances individuals’ levels of self-acceptance, ultimately leading to a reduction in mobile phone addiction tendencies. In other words, physical activity is associated with lower levels individuals’ subjective experience of stress, which may be linked to a more a positive recognition of self-worth and reflect a potential pathway extending from external behavioral regulation to internal psychological restoration.

This finding deepens our understanding of the mechanisms through which physical activity intervenes in addictive behaviors, from the perspective of dynamic psychological processes. It reveals that the effects of physical activity are not limited to emotional catharsis or behavioral substitution, but also involve a systemic restructuring of individuals’ internal regulatory capacities. Enhanced emotional regulation enables individuals to respond to stressors with greater resilience and self-control, while improved self-acceptance is associated with a more stable psychological structure and a diminished reliance on external validation. Consequently, individuals exhibit a lower tendency to use mobile phones as a means of escaping reality or seeking virtual comfort.

## 5 Limitations

Despite the encouraging results, this study is subject to several limitations that should be acknowledged. First, the use of a cross-sectional research design restricts the ability to establish causal relationships among variables. This methodological constraint limits the depth of causal inference and hinders the understanding of temporal dynamics. Second, data were obtained exclusively through self-reported questionnaires, which may be affected by social desirability bias and recall inaccuracies. Such biases can compromise the objectivity and precision of the findings. Third, the sample was solely drawn from Xuzhou Medical University, thereby potentially limiting the external validity and generalizability of the conclusions. The participants represent a specific institutional and regional cohort, and extrapolation to other student populations or broader demographics should be approached with caution. Fourth, the reliance on self-administered questionnaires may have introduced common method bias. Although the Harman single-factor test was conducted to assess this concern, its limitations necessitate the use of more robust procedural remedies in future research, such as counterbalancing the order of measurements and ensuring anonymity to reduce response bias. Finally, although this study provided preliminary insights into the relationship between physical activity and smartphone addiction, it did not fully explore the differential mechanisms underlying various types of physical activity, nor did it examine the potential moderating role of gender. Future research should build upon work to further investigate these underlying mechanisms, thereby enhancing the scientific rigor, specificity, and practical relevance of intervention strategies aimed at mitigating problematic smartphone use (Du and Zhang, 2022, Zhihao et al., 2024).

## 6 Implications for research

To address the aforementioned limitations and extend the current findings, several avenues for future research are proposed. First, adopting longitudinal or experimental study designs would allow for more rigorous examination of causal relationships and temporal mechanisms. Second, the incorporation of objective behavioral monitoring tools—such as mobile device usage logs and physiological indicators—would enhance data accuracy and ecological validity. Third, future studies should seek to include more diverse and representative samples from various geographic regions, cultural backgrounds, and age groups, thereby improving the generalizability and cross-contextual applicability of the model. Fourth, future research should further explore the moderating and mediating mechanisms of different types and intensities of physical activity in relation to smartphone addiction. Particular attention should be given to the differential effects of various forms and intensities of aerobic exercise on alleviating problematic smartphone use, as well as the underlying psychological and neurophysiological mechanisms involved. Moreover, it is essential to systematically examine the potential moderating role of gender in the relationship between physical activity and Internet addiction, in order to elucidate how individual differences influence intervention outcomes and to provide a theoretical foundation for developing more targeted and effective prevention and intervention strategies. Lastly, drawing inspiration from recent methodological advancements such as the machine learning approach employed (Zang et al., 2025), future research may benefit from utilizing nonlinear modeling techniques to capture the complex interactions among variables, offering more nuanced insights beyond the capacity of traditional statistical methods.

## 7 Conclusion

This study identified a significant negative association between physical activity and mobile phone addiction among university students, with perceived stress and self-acceptance serving as mediating factors. These findings contribute to a more nuanced understanding of the potential pathways linking physical activity with mobile phone use behaviors. Nevertheless, the cross-sectional design of this research limits the ability to draw causal inferences. The observed associations should therefore be interpreted as correlational rather than causal in nature.

The results highlight the importance of considering psychological factors such as perceived stress and self-acceptance when examining behavioral patterns related to mobile phone use. Future longitudinal and experimental research is warranted to further clarify these relationships and to determine whether physical activity and psychological factors interact in ways that may influence mobile phone use over time.

In conclusion, this study provides preliminary insights into the interplay between physical activity, perceived stress, self-acceptance, and mobile phone addiction among university students. While the findings suggest possible directions for future inquiry, further evidence is needed before firm conclusions can be drawn or intervention strategies can be developed.

## Data Availability

The raw data supporting the conclusions of this article will be made available by the authors, without undue reservation.
